# Where Is the Bullet? A Puzzling Case of an Untraceable Projectile in a Gunshot Injury

**DOI:** 10.7759/cureus.67188

**Published:** 2024-08-19

**Authors:** Toshal Wankhade, Ravi Kumar Sharma, Amit Patil, Ashok Kumar Rastogi, Nikhil Goel

**Affiliations:** 1 Forensic Medicine and Toxicology, All India Institute of Medical Sciences, Patna, Patna, IND; 2 Forensic Medicine and Toxicology, IQ City Medical College, Durgapur, IND

**Keywords:** bullet exit, bullet trajectory, bullet embolism, missing bullet, gunshot wound

## Abstract

In forensic examinations, gunshot injury cases can sometimes present unusual challenges. Typically, a gunshot injury involves an entry wound where the bullet penetrates the body and an exit wound where the bullet exits. If the bullet does not exit the body, it can often be recovered from the body cavity. However, there are instances where the entry wound is identified, but both the exit wound and the bullet appear to be missing. This paper explores such a paradox, where, despite a thorough postmortem examination, neither the bullet nor the exit wound is found. We consider various possibilities and analyze whether this was indeed a gunshot injury. Could the bullet still be inside the body, or might it have taken an unexpected route? This paper aims to clarify this puzzling phenomenon through detailed analysis and underscore the importance of meticulous forensic investigation in complex cases.

## Introduction

Gunshot injuries are a leading cause of mortality worldwide, with alarming trends showing a consistent increase in their occurrence [1]. In India, especially in the northern states, firearm homicides are a common form of assault, exacerbated by the widespread availability of weapons through illicit trade. Most fatalities from firearm injuries are linked to locally manufactured weapons [2,3]. Gunshot injuries present diverse patterns in terms of injury manifestation and bullet trajectory [4].

A key objective of postmortem examinations for firearm injuries is to recover the bullet during the autopsy. This bullet is crucial for forensic investigations as it helps trace the weapon used, identify the perpetrator, and reconstruct the crime scene [4,5]. Typically, a gunshot wound includes an entry wound, a bullet trajectory within the body, and an exit wound where the bullet leaves the body. Understanding the trajectory and recovering the bullet is essential for forensic investigations into gunshot wounds [5,6].

Autopsy surgeons use their experience and knowledge to offer medicolegal opinions based on the pattern of wounds and trajectory in gunshot cases. Sometimes, however, a patient may present with an entry wound but no corresponding exit wound, and locating the bullet can be challenging due to its unconventional path. In such cases, pinpointing the bullet’s location often requires thorough exploration, with radiological imaging playing a crucial role in locating and recovering the projectile [7].

If there is no exit wound and the bullet remains elusive despite exhaustive searches, the question arises: where could the bullet be? This paper discusses a unique case of a fatal firearm injury with an entry wound but no identifiable bullet or exit wound, presenting a challenging forensic scenario. Various hypotheses about potential exit routes, including the possibility of the bullet exiting through natural orifices, are considered. This analysis is supported by relevant literature.

## Case presentation

The case involved an individual who sustained a firearm injury. According to the alleged history, the deceased, a lorry driver, was driving his truck around 01:00-01:15 hours at midnight on the highway. The driver was stopped midway by robbers on a motorcycle. One of the robbers fired a bullet from a country-made firearm into the right side of the driver’s chest. After the shooting, the robbers pulled him out of the truck and fled with the vehicle. The driver managed to crawl to the nearest shop and called the shop owner. The shop owner and nearby people informed the police and took the injured driver to the nearest hospital, where he was admitted and treated with ionotropic support. He remained hospitalized until 16:00 that day. When his relatives arrived, he was referred to our institute for further management and reached our hospital around 18:00 hours, approximately 17 hours after the incident. His relatives reported that he had passed stool during his earlier hospital stay.

According to the clinical notes from our institute, the deceased had a bullet entry wound on the right side of the chest at the time of admission. He was conscious and well oriented, and he recounted the details of the incident himself. During his hospital stay, radiological investigations, including a chest and pelvis X-ray and a CT scan of the abdomen and thorax, were performed. The radiologists reported that no bullet was visible inside the body cavity. The CT scan noted an entry wound on the right anterior chest wall but did not reveal an exit wound.

**Figure 1:**
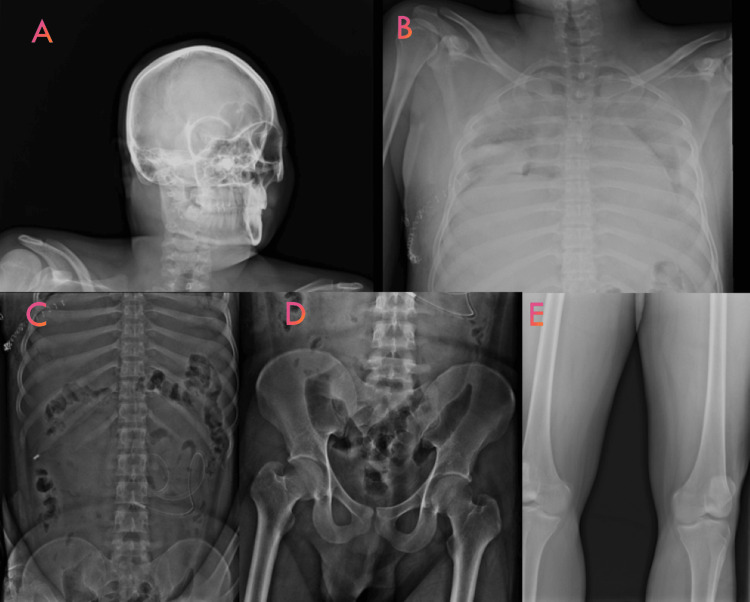
X-ray of the entire body from head to toe (A: head, B: neck and chest cavity, C: abdominal cavity, D: pelvic cavity, and E: lower limb) performed prior to the autopsy shows no evidence of a bullet

The patient underwent exploratory laparotomy and thoracotomy the following day. The operative notes indicated multiple intestinal perforations: one at the pylorus of the stomach (4 cm × 3 cm), two on the jejunum approximately 100 cm distal to the duodenojejunal junction (2 cm × 1 cm and 1 cm × 1 cm), and one on the colon 100 cm from the ileocecal junction (1 cm × 1 cm). The perforations were sutured, and a drain was placed in the abdominal cavity. The operating surgeon’s notes indicated that no bullet was recovered during the surgical procedure. The patient’s condition deteriorated, and he died the day after the operation. His total hospital stay at our institute was 36 hours. The body was later sent for postmortem examination at our departmental mortuary by the police. During the postmortem examination, following our institute’s autopsy protocol, the body was subjected to an X-ray before the autopsy. The X-ray did not reveal the presence of any bullets inside the body cavity (Figure 1).

**Figure 2:**
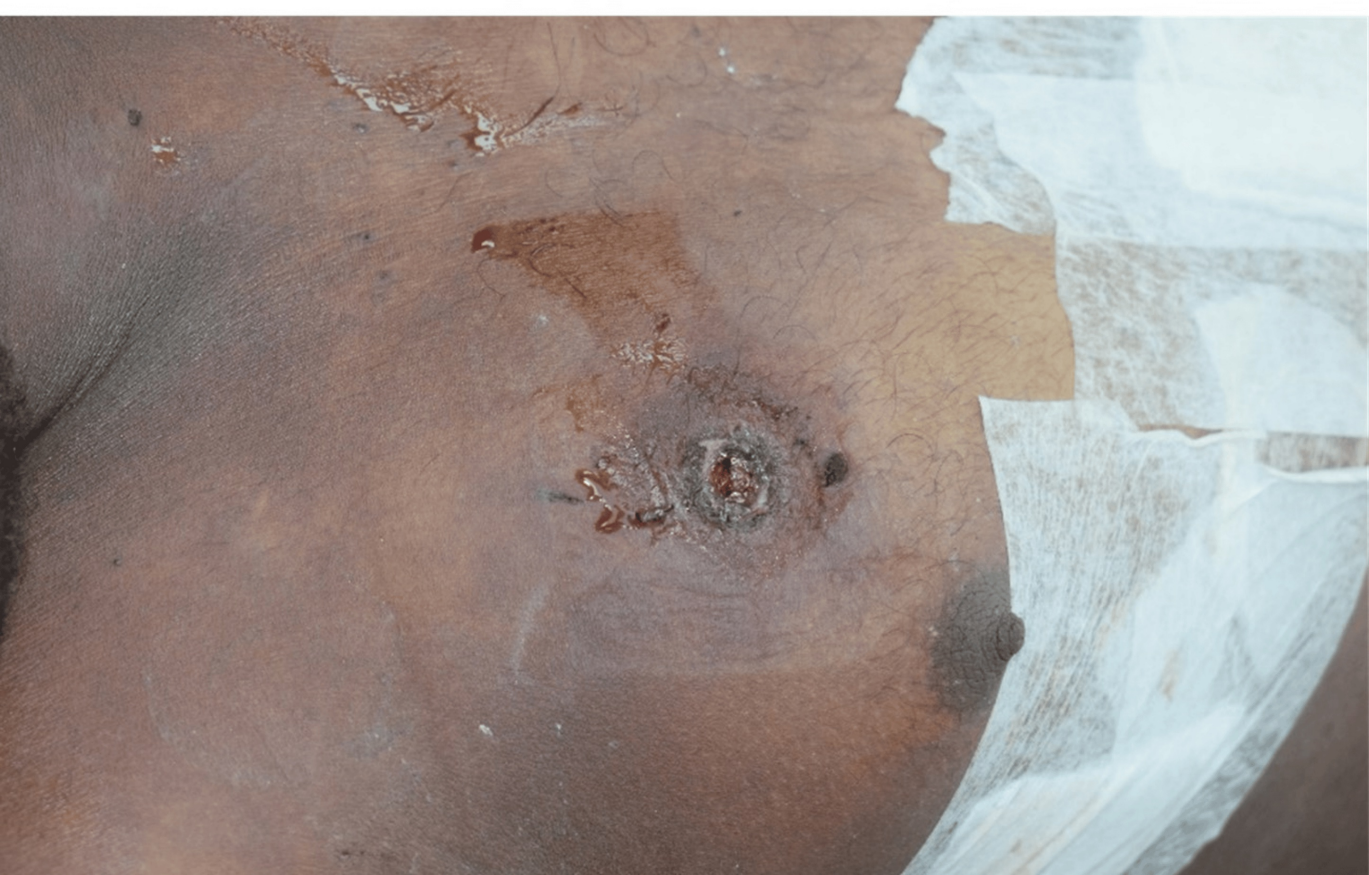
Firearm entry wound with surrounding abrasion collar on the right anterior chest

On external examination during the postmortem, there was a firearm entry wound with the following characteristics: dimensions of 1.5 cm × 1 cm × depth into the thoracic cavity, with inverted margins, located on the right side of the chest in the third intercostal space. An abrasion collar surrounded the wound (Figure 2).

The projectile entered the right thoracic cavity, contacting the upper border of the fourth right rib at its costochondral junction. It penetrated the lower border of the right lung medially and continued into the abdominal cavity by perforating the right dome of the diaphragm. Surgical repair of the damaged diaphragm was evident, with one blue-colored intact suture.

**Figure 3:**
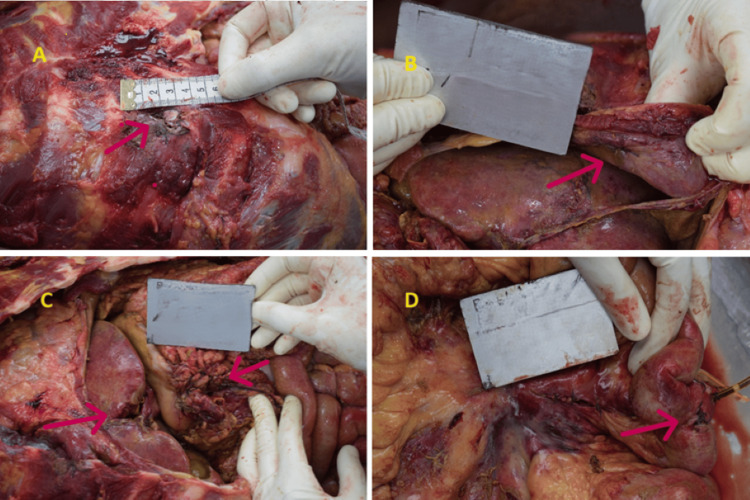
Projectile track in the chest and abdominal cavity with damage to various organs observed during autopsy: (A) Bullet entering the chest cavity. (B) Bullet piercing the diaphragm with visible surgical repair. (C) Bullet tearing the liver and entering the abdominal cavity. (D) Damage caused by the bullet in the intestine

The projectile’s path continued with a penetrating laceration of the left lobe of the liver, located 1 cm lateral to the falciform ligament. It also perforated the wall of the stomach at its pyloric end, with a surgically repaired wound at the pylorus. The projectile then perforated the mesentery of the small intestine, creating a wound in the jejunum, which was also surgically repaired. Finally, the bullet entered the large intestine, as indicated by a surgically repaired wound 100 cm away from the ileocecal junction in the descending colon (Figure 3).

Despite a meticulous autopsy and thorough search, no projectile was recovered from the abdomen.

## Discussion

After a thorough postmortem examination failed to reveal the bullet, the key question remained: “Where is the bullet?” We explored various possibilities, beginning with confirming whether it was indeed a gunshot injury. However, the characteristics of the wounds - such as the typical entry wound with an abrasion collar, the trajectory of the projectile within the body cavity, and the deceased’s account of the incident - clearly indicated that it was a gunshot wound.

The possibility of the bullet still being inside the body cavity was ruled out through a thorough postmortem examination and complete radiological imaging conducted before the autopsy. We also considered whether the bullet might have been removed during surgery, but this seemed unlikely. Radiological exams, including X-rays and CT scans, did not show the presence of a bullet before surgical interventions. Additionally, the bullet was not recovered during the surgery performed by the treating surgeon. The CT scan report confirmed that it was a case of a gunshot entry wound without an exit wound, and no bullet was found inside the body cavity.

Another possibility to consider is that the bullet may have taken an unexpected route. Literature suggests that bullets can follow unusual pathways within the body. For instance, bullets may enter the stomach and be vomited out, enter the windpipe and be coughed out, or enter the mouth and be spat out. They may also exit through the same entry wound after striking a bone or migrate into the gastrointestinal tract and be expelled through feces [4,8]. In this case, given the perforation found in the large intestine 100 cm distal to the ileocecal junction, it is plausible that the bullet entered the gastrointestinal tract and exited through this route. To support this hypothesis, the relevant literature has been thoroughly reviewed.

Haghiri et al. suggest in their case report that bullets entering the intestine can exit through the body’s natural orifices [9]. Krispin et al. described a case where a bullet, after penetrating the abdominal cavity, entered the intestinal lumen and initially halted there. The bullet then migrated within the intestine, a phenomenon Krispin et al. termed “intestinal embolism of the bullet” [10]. Similarly, Biswas et al. reported a rare case of gastrointestinal bullet embolism, where a small-caliber bullet perforated the small bowel, migrated distally, and eventually lodged in the distal colon [11]. Other reports detail bullets migrating from the esophagus to the stomach [12,13], or exiting through the mouth [14]. There is also a case where a bullet entered the chest cavity and was expectorated by the hemodynamically stable patient without surgical intervention [15].

These cases highlight the diverse possible trajectories of bullets in gunshot wounds. Forensic experts should consider all potential variations when performing autopsies on firearm cases. In this instance, we hypothesize that the bullet may have exited through the gastrointestinal tract, given the absence of an exit wound and the failure to recover the bullet. To support this hypothesis, we reviewed the patient’s medical records, the detailed history provided by the next of kin, radiological examinations, and clinical notes from the treating surgeon. This hypothesis aligns with existing literature on similar occurrences, emphasizing its plausibility.

Ultimately, considering the deceased’s history, medical records, preoperative radiological imaging, operative notes, pre-autopsy X-ray investigations, and detailed autopsy examination, it is likely that the projectile exited through the gastrointestinal tract while the patient was alive. The peristaltic movement of the gastrointestinal tract may have propelled the projectile, leading to its expulsion through the stool prior to the patient’s admission. This fact was not evident while the patient was alive.

## Conclusions

Although uncommon, the possibility of a bullet exiting through a natural orifice is supported by the findings in this case. It underscores the need for careful consideration of various scenarios and an understanding of atypical bullet trajectories in forensic examinations of gunshot injuries. This case also highlights the importance of interdisciplinary collaboration and detailed analysis to resolve complex forensic medical cases. It involved thorough radiological investigations, input from the treating surgeon and radiologist, and a conclusion drawn from these contributions. From a forensic expert’s viewpoint, meticulous documentation of findings, encompassing radiological reports, surgical notes, and autopsy findings, is a keystone for framing accurate and reliable forensic opinions.
